# Affective Cognition of Students’ Autonomous Learning in College English Teaching Based on Deep Learning

**DOI:** 10.3389/fpsyg.2021.808434

**Published:** 2022-01-19

**Authors:** Dian Zhang

**Affiliations:** ^1^College of Foreign Languages, Hunan University, Changsha, China; ^2^College of Foreign Languages, Hunan University of Humanities, Science and Technology, Loudi, China

**Keywords:** cognitive activities, English learning, deep learning, emotional interactions, mining learning

## Abstract

Emotions can influence and regulate learners’ attention, memory, thinking, and other cognitive activities. The similarities and differences between English and non-English majors in terms of English classroom learning engagement were compared, and the significant factors affecting the emotional, cognitive, and behavioral engagement of the two groups of students in the English classroom were different. English majors’ affective engagement in the classroom was not significant, which was largely related to their time and frequency of English learning. Traditional methods of learner emotion recognition suffer from low recognition rate, complex algorithms, poor robustness, and easy to lose key information about facial expression features. The paper proposes a convolutional neural network-based learner emotion recognition method, which includes three convolutional layers, three pooling layers, and one fully connected layer. In the future, the method will can be applied to the construction of smart learning environments, providing technical support for improving learner models, realizing emotional interactions, and mining learning behaviors, etc.

## Introduction

According to the Guide to Teaching English in College 2020, “College English,” as a compulsory course in higher education, is both humanistic and instrumental, and plays an important role in improving the overall quality of college students (Ahltorp et al., 2016; Wang, 2016). The English language course should be teacher-oriented, mobilize students’ motivation to learn foreign languages, and meet the future development needs of the country and students (Lee and Park, 2008). However, in the foreign language teaching environment, learning engagement is an important indicator to measure the learning process and influence the learning effect (Su et al., 2013). In recent years, foreign language education scholars in China have also begun to focus on the study of students’ learning engagement. Therefore, the author follows the research trend and compares students’ learning engagement in English classrooms with students of other languages, aiming to provide reference for English teaching practice, research and reform in China.

Smart learning environments focus on the development of higher-order skills such as innovation, problem solving, decision making, and critical thinking, where cognitive activities play a critical role in coordinating and controlling the development process (Lu and Fan, 2021).

Cognitive activities play a critical role in the development process (Luo et al., 2012). Emotions are psychological responses caused by external stimuli that influences and regulates cognitive activities such as attention, perception, representation, memory, thinking, and language (Irawan et al., 2020; Pazyura et al., 2020).

The 2016 Horizon Report (Higher Education Edition) states that Affective Computing (ACE) will be commonly used in the next 4–5 years (Jain et al., 2016; Zhao and Qin, 2021). It is evident that facial expressions play a very crucial role in the way learners express their emotions. And in practice, it is more natural and feasible to capture the learner’s facial expressions through the learning device’s own camera, and then identify the learner’s emotional state than other methods (Xie et al., 2018; Juárez-Díaz and Perales, 2021; Zhang, 2021).

With the rapid development of technology, deep learning (DL) has become an important machine learning algorithms in artificial intelligence. DL combines image feature extraction with fuzzy classification by neural networks, omitting the complex image pre-processing and feature extraction process at the front end, making it no longer dependent on manually elaborated explicit feature extraction methods, which improves the performance, generalization ability and also the robustness of recognition algorithms (Dao and Sato, 2021). In this study, we construct our own large-scale learner emotion database and propose a DL-based learner emotion recognition method in order to improve the efficiency and accuracy of learner emotion recognition, provide technical support for harmonious emotional interaction in smart learning environments, and promote easy, engaged, and effective learning for learners.

## Related Work

Deep learning is the expression of individual students’ active participation in the learning process, deep thinking, active problem solving, and positive emotional experience (Ahltorp et al., 2016), and is an important predictor of student learning outcomes and achievement (Judy Shih, 2021). Among them, behavioral, affective, and cognitive inputs are the main components of learning engagement, and affective and cognitive input is important foundations of behavioral input. In recent years, scholars at home and abroad have conducted a lot of research around the topic of student engagement (Webb et al., 2021). Different dimensions of students’ learning motivation, such as interest in learning and self-efficacy, were maintained at high levels; teacher feedback, teaching environment, and interpersonal relationships acted as mediators of learning motivation and learning engagement, which in turn had an impact on learning motivation (Zhang et al., 2020; Thomas et al., 2021). It was also found that learning engagement was related to students’ individual differences, such as learning motivation self, in the blended teaching model. Xin (2021) found that the second language motivational self had a positive effect on learning engagement, but anxiety had a negative effect on learning engagement by constructing a model of college students’ English learning engagement. In addition, some studies, such as Pazyura et al. (2020), have further analyzed the correlation between learning engagement and academic performance, but comparative studies on learning engagement among different groups of students are still lacking. However, learning engagement in foreign language classrooms must involve students’ cognitive level, affective state, and behavioral performance (Chen et al., 2021; Lu and Fan, 2021).

Emotion is the experience of people’s attitudes toward objective things in social activities, and it is a special form of psychological reflection of objective things, which play an important role in people’s thought perception and behavior performance (Zhang et al., 2019; Marantika, 2021). Deng (2021) constructed SLE-FER, an emotion analysis framework based on facial expression recognition based on Facial Action Coding System (FACS) proposed by Aikman, including perception layer, transmission layer, data layer, analysis layer, and application layer, and used tensor decomposition algorithm for expression recognition. Cao et al. (2021) combined eye-tracking and facial expression recognition and proposed an intelligent agent-based emotion and cognition recognition model for distance learners, which couples eye-tracking with iterative recognition of expression monitoring and emotional and cognitive processes to improve recognition accuracy. Hou (2021) proposed FILTWAM, a framework for improving learner learning through webcam head and Microwind, which recognize learner emotions and provides timely feedback based on learners’ facial expressions and verbal expressions. Wu et al. (2021) proposed a model of student emotion recognition based on cognitive evaluation based on the theory of OCC model, used fuzzy inference method to realize the expectation degree inference of learning events, and tested and evaluated the constructed model by constructing a dynamic Bayesian network for computer simulation (Przymuszała et al., 2021).

To sum up, scholars at home and abroad have conducted extensive research on learner emotion recognition, among which the most research on learner emotion recognition based on facial expressions has been conducted. Wang et al. (2018) and Karimova and Csapó (2021) pointed out that recognition methods based on facial expressions in education are more usable than other emotion recognition methods. However, most of the current studies use traditional machine learning methods of facial recognition, feature extraction, feature selection, and training classification, which are inefficient and difficult to guarantee whether the manually selected features can effectively reflect facial expressions. Therefore, this study uses CNN with autonomous learning capability to achieve effective recognition of learners’ emotions.

## Our Model

As an important method of DL, CNN has the features of weight sharing and local connectivity, which reduce the complexity of the network and facilitates parallel processing (Golparvar and Tabatabaie Nejad, 2021). The feedforward operation stage of CNN extracts the high-level semantic information of raw data such as image and audio layer by layer through a series of operations. The different types of operations are generally referred to as layers, i.e., convolutional operations, and pooling operations, i.e., pooling layers. CNN usually includes input layer, convolutional layer, pooling layer, fully connected layer, and output layer.

### Convolutional Layer

The convolutional layer is the feature extraction layer, which is the basis of CNN. Each convolutional layer consists of multiple neurons, and each neuron is composed with all the feature maps of the previous layer using multiple trainable convolutional kernels, respectively, plus bias values, which are solved as parameters of the activation function, and the output values will form the new feature image (Li et al., 2016). The convolution kernel size and convolution step size is important adjustment parameters. The convolution layer is calculated as:


(1) $${\text{y}}_{\text{j}}^{1}=\text{f}\,\left(\sum_{\text{i}=1}^{{\text{N}}^{1-1}}{\text{y}}_{\text{i}}^{1-1}⊗{\text{w}}_{\text{ij}}^{1}+{\text{b}}_{\text{j}}^{1}\right)$$

In Equation 1, l denotes the current layer; l−1 denotes the previous layer; f() is the activation function.

The commonly used activation functions in CNN are linear correction function (ReLU), hyperbolic tangent S-shaped function (Tanh), logarithmic S-shaped function (Sigmoid), etc. The activation function used in this study is sigmoid function (Zhang, 2021).

### Pooling Layer

The pooling layer is also known as the down sampling layer. The number of feature maps will increase due to the incremental increase in the number of convolutional layers, resulting in the number of learned feature dimensions will grow rapidly and cause difficulties for the classifier. The key role of the cooling layer is to reduce the computational effort and the number of parameters, and to prevent overfitting to a certain extent, making it easier to optimize. Pooling layer does not change the number of feature maps, but makes the size of the feature maps smaller. There are two main types of pooling: mean pooling and maximum cooling. The type of pooling operation, kernel size, and step size are important adjustment parameters, and this study uses mean pooling. The pooling layer is calculated as:


(2) $${\text{y}}_{\text{j}}^{1}=\text{f}\,\left(\beta_{\text{j}}^{1}\,\text{down}\,\left({\text{y}}_{\text{j}}^{1-1}+{\text{b}}_{\text{j}}^{1}\right)\right)$$

In Equation 2, down() denotes the pooling function ${\text{y}}_{\text{j}}^{1}$ and ${\text{y}}_{i}^{1-1}$ denote the j-th feature image of the current layer and the previous layer; $\beta_{\text{j}}^{1}$ and ${\text{b}}_{\text{j}}^{1}$ denote the weight coefficients and bias values of the j-th feature image of the current layer.

### Fully Connected Layer

The fully connected layer acts as a “classifier” in the whole CNN, and the output of the fully connected layer is used as the input to the output layer or the final classification result, and CNNs usually have one or more fully connected layers. Each neuron of a fully connected layer will be connected to all neurons in the previous layer, combining the features extracted from the convolutional and pooling layers (Zhang et al., 2019). In practice, fully connected layers can be implemented by convolutional operations, and a fully connected layer for which the previous layer is fully connected can be transformed into a convolution with a convolution kernel of 1 × 1; while a fully connected layer for which the previous layer is a convolutional layer can be transformed into a global convolution with a convolution kernel of h × w. h and w are the height and width of the convolutional output of the previous layer, respectively.

## Convolutional Neural Network

**FIGURE 1:**
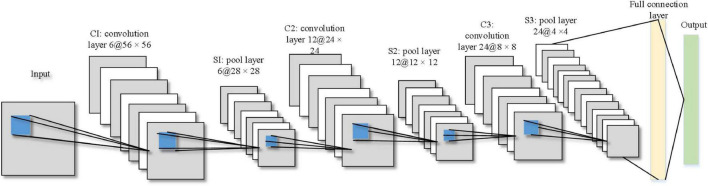
Structure of seven-layer CNN.

The design of CNN structure needs to consider three factors, such as accuracy, training speed, and memory consumption. Small convolutional kernels can increase the network depth and reduce the number of parameters. Usually, the convolutional kernel size is set to 3 × 3 or 5 × 5, and in this study, the convolutional kernel size is set to 5 × 5 with a step size of 1. The low number of layers in the network will lead to insufficient information representation, and increasing the number of layers in the network will lead to gradually stronger feature information representation, but too many layers will also lead to an overly complex network structure, increased training time, and overfitting (Marantika, 2021). The input image size, convolutional kernel size, convolutional step, pooling window size, and pooling step jointly determine the number of layers in the network. In this study, a seven-layer CNN is designed based on the characteristics of the learner’s facial expression images, including three convolutional layers, three pooling layers, and one fully connected layer, and the structure is shown in Figure 1.

(1) The input layer is a 60 × 60 pixel images of the learner’s facial expressions. (2) In the C1 layer, the input image is convoluted with six 5 × 5 convolution kernels with a step size of 1 and the activation function is a Sigmoid function. It is mainly through one filter to continuously extract features, from local features to overall features, so as to carry out image recognition and so on.

## Parameter Training of Convolutional Neural Networks

Training of CNN is to the internal parameters of CNN using the sample set of facial expressions that have been labeled with emotional types and intensities. The number and quality of the samples directly determines the performance and generalization ability of DL. In this study, the types of learner emotions are firstly defined, and then a large-scale learner emotion database is constructed and used as the training sample set to train the CNN, so as to achieve accurate recognition of learner emotions.

### Learner Emotion Database

Emotion recognition based on facial expressions cannot be achieved without the support of expression databases. At present, emotion recognition research begins to develop for various professional fields, especially learner emotion recognition has received wide attention from researchers, but learner emotion databases constructed based on facial expressions are not common (Su et al., 2013). Therefore, construction of a learner emotion database based on facial expressions has a certain promotion effect on the in-depth research of learner emotion recognition algorithms.

Types of learner emotions: emotion is the experience of human attitudes toward objective things and corresponding behavioral reactions. For the description of emotion, there are “seven emotions and six desires” in ancient China, while the Western thinker Rene Descartes believed that there are six primitive emotions. At present, the field of psychology mainly focuses on two theories of emotion classification: basic emotion and dimensional emotion. Based on the study of facial expressions and behavioral responses, classified the basic emotions into pleasure, surprise, sadness, anger, fear, and disgust (Cao et al., 2021; Deng, 2021). Przymuszała et al. (2021) argued that emotions have three dimensions of intensity, similarity, and bipolarity, and he used the image of an inverted cone to describe the relationship between the three dimensions, with eight cross-sections representing the eight basic emotions of ecstasy, acceptance, surprise, fear, grief, hatred, rage, and vigilance, respectively.

Learners’ emotions are unique, although they have universal characteristics of human emotions. Sun Bo summarized learners’ emotional types as happy, surprised, bored, confused, tired, focused, and confident (Wang, 2016).

Building a database of learner emotions: at present, the main databases of facial facial expressions are the Japanese Female Facial Expression Database (JAFFE), the CK (Cohn-Kanade) Facial Expression Database and its extension database CK+ Facial Expression Database of Carnegie Mellon University, the University of Maryland Facial Expression Database, the Tsinghua University Facial Expression Database, the CED-WYU Facial Expression Database of Wuyi University, the BNU Learning emotion database, etc. (Przymuszała et al., 2021). First, because the face and expressive features of foreigners are significantly different from those of the Chinese people, it is difficult to generalize the training results in China by using foreigner’s facial expression database for training. Secondly, the number of samples in the known facial expression database is small and most of them are adults, which are difficult to meet the need of DL and practical applications. Finally, it is difficult to obtain facial expression databases from other institutions or organizations due to privacy protection. Therefore, this study chooses to build a learner emotion database based on facial expressions independently.

The subjects were 70 graduate students, 18 males and 52 females, ranging in age from 20 to 29 years. Before the formal acquisition of expressions, they were trained in groups so that they could present standard types of emotions and intensities in their natural state as much as possible. The acquisition platform was written in C++ and the acquisition device was a high-definition camera. When formally capturing expressions, each graduate student showed seven types of emotions, including normal, happy, angry, sad, frightened, focused, and bored, and each emotion showed five intensities from weak to strong, capturing 30 images for each intensity, forming a raw database with 73,500 images of learners’ facial expressions. For example, in 0001_02_03_0004, 0001 indicates the subject number, 02 indicates the emotional type, 03 indicates the emotional intensity, and 0004 indicates the image number.

**FIGURE 2:**
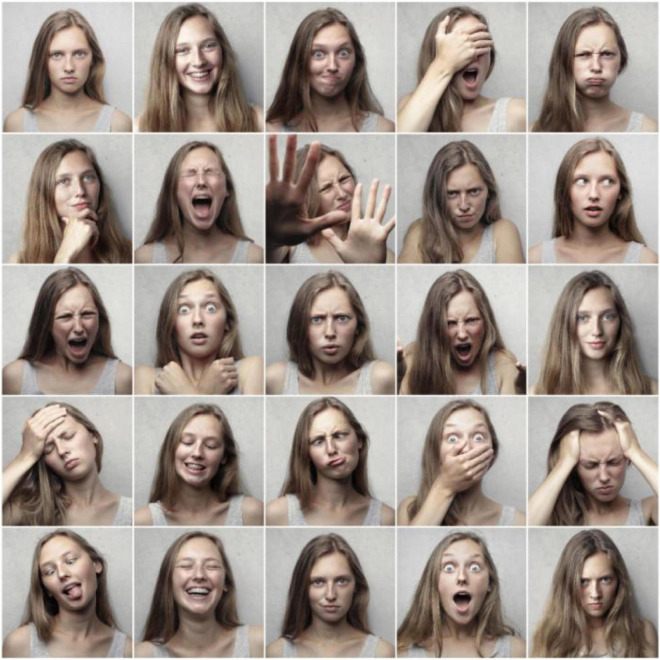
Sample training sample set of different emotions.

Then, we used the AdaBoost method based on Haar rectangle feature to detect the faces of 73,500 images in the original database and extracted a total of 70,090 face images. The face detection algorithm is relatively mature and complete, so we will not repeat it in this article. The background is complicated because of the dormitory and study room in the early stage of acquisition, which causes problems for face detection. In the later stage, a solid color background was used for acquisition, and the face detection accuracy was higher. Finally, 60,000 facial expression images were selected as training samples for DL and 9,000 facial expression images were selected as test samples for DL in this study. Sample facial expressions of different emotions are shown in Figure 2.

## Research Data Analysis

### Regression Analysis of Two Types of Students’ Engagement in English Classroom Learning

A stepwise regression analysis was conducted with teacher feedback, teaching attitude, learning motivation, learning resources, learning regulation, group work, teacher–student interaction, and English knowledge objects as independent variables and behavioral input as dependent 0.269 for group work (*t* = 4.359, *p* = 0.000 < 0.01), 0.203 for teacher–student interaction (*t* = 3.195, *p* = 0.002 < 0.01), and 0.128 for English knowledge (*t* = 2.255, *p* = 0.026 < 0.05). Therefore, motivation, learning control, group work, teacher–student interaction, and English knowledge had a significant positive effect on non-English majors’ behavioral engagement in English classes and were the specific factors that influenced non-English majors’ behavioral engagement in English classes (Table 1).

**TABLE 1:** Stepwise regression results of factors influencing behavioral input of non-English majors.

	Non standardized coefficient		Standardization coefficient	*t*	*p*	VIF	*R* ^2^	Adjustment *R*^2^	*F*
	*B*	SE	Beta						
Constant	0.125	0.070	–	1.788	0.077	–			
Learning motivation	0.191	0.051	0.209	3.749	0.000	10.572			
Learning regulation	0.174	0.049	0.199	0.535	0.001	10.857			
Group cooperation	0.269	0.062	0.273	4.359	0.000	13.297	0.971	0.969	*F*(5,99) = 659082, *p* = 0.000
Teacher–student interaction	0.235	0.064	0.201	3.197	0.002	13.443			
English knowledge	0.128	0.057	0.132	2.255	0.026	11.570			

Similarly, stepwise regression analysis was conducted with teacher feedback, teaching attitude, learning motivation, English knowledge, learning resources, learning control, group work, and student–teacher interaction as independent variables and behavioral input as dependent 0.109 for learning moderation (*t* = 2.511, *p* = 0.014 < 0.05), 0.214 for group work (*t* = 4.042, *p* = 0.000 < 0.01), and 0.201 for teacher–student interaction 2 (*t* = 3.939, *p* = 0.000 < 0.01). Therefore, motivation, learning resources, learning regulation, group work, and student–teacher interaction had significant positive effects on English majors’ classroom behavioral engagement and were specific factors that influenced English majors’ English classroom behavioral engagement (see Table 2).

**TABLE 2:** Stepwise regression results of factors influencing behavioral engagement of English majors.

	Non standardized coefficient		Standardization coefficient	*t*	*p*	VIF	*R* ^2^	Adjustment *R*^2^	*F*
	*B*	SE	Beta						
Constant	0.042	0.061	–	0.690	0.177	–			
Learning motivation	0.172	0.042	0.188	4.067	0.000	9.858			
Learning regulation	0.291	0.045	0.291	6.487	0.000	9.658			
Group cooperation	0.109	0.043	0.124	2.511	0.014	11.299	0.979	0.977	*F*(5,99) = 901.940, *p* = 0.000
Teacher–student interaction	0.214	0.053	0.217	4.042	0.000	13.31			
English knowledge	0.201	0.051	0.199	3.939	0.000	11.117			

A stepwise regression analysis was conducted with teacher feedback, teaching attitude, learning motivation, learning resources, learning regulation, group work, teacher–student interaction, and English knowledge as independent variables and affective engagement as dependent variables, with regression coefficient values of 0.091 for teacher feedback (*t* = 2.182, *p* = 0.031 < 0.05), 0.190 for teaching attitude (*t* = 3.445, *p* = 0.001 < 0.01), 0.137 for motivation (*t* = 2.448, *p* = 0.016 < 0.05), 0.212 for learning resources (*t* = 4.219, *p* = 0.000 < 0.01), 0.148 for learning regulation (*t* = 3.372, *p* = 0.001 < 0.01) The regression coefficient value for teacher–student interaction was 0.198 (*t* = 3.707, *p* = 0.000 < 0.01). Therefore, teacher feedback, teaching attitudes, motivation, learning resources, learning regulation, and teacher–student interaction had significant positive effects on German majors’ emotional engagement in English classes and were the specific factors that influenced German majors’ emotional engagement in English classes (Table 3).

**TABLE 3:** Stepwise regression results of factors influencing emotional engagement of non-English majors.

	Non standardized coefficient		Standardization coefficient	*t*	*p*	VIF	*R* ^2^	Adjustment *R*^2^	*F*
	*B*	SE	Beta						
Constant	0.100	0.062	–	1.614	0.110				
Learning motivation	0.091	0.042	0.098	2.182	0.031				
Learning regulation	0.190	0.055	0.195	3.445	0.001	13.684			
Group cooperation	0.137	0.056	0.148	2.488	0.016	15.536	0.977	0.976	*F*(5,99) = 694.957, *p* = 0.000
Teacher–student interaction	0.212	0.050	0.222	4.219	0.000	11.778			
English knowledge	0.148	0.044	0.167	3.372	0.001				

The regression coefficient value for teacher–student interaction was 0.168 (*t* = 3.161, *p* = 0.002 < 0.01). Therefore, teacher feedback, teaching attitude, motivation, English knowledge, learning resources, learning regulation, and student–teacher interaction have significant positive effects on affective engagement and are specific positive factors that affect non-English majors’ affective engagement in the classroom (Table 4).

**TABLE 4:** Results of specific factors affecting in English classroom.

	Non standardized		Standardization	*t*	*p*	VIF	*R* ^2^	Adjustment *R*^2^	*F*
	*B*	SE	Beta						
Constant	0.06	0.02	–	1.01	0.35				
Learning motivation	0.15	0.51	0.11	2.64	0.10	1.775			
Learning regulation	0.47	0.04	0.18	3.29	0.00	1.787			
Group cooperation	0.14	0.05	0.87	3.46	0.01	13.67	0.977	0.96	*F*(5,9) = 69.587, *p* = 0.02
Teacher–student interaction	0.18	0.09	0.15	3.05	0.03	7.95			
English knowledge	0.18	0.53	0.63	3.15	0.02	1.358			

### A Path Analysis of Two Types of Students’ Engagement in English Classroom Learning

According to the results of the above regression analysis, for non-English majors, I generated the first-level variables, i.e., behavioral input, from learning motivation, learning regulation, group cooperation, teacher–student interaction, and English knowledge; and the first-level variables, i.e., affective input, from teacher feedback, teaching attitude, learning motivation, learning resources, learning regulation, and teacher–student interaction; and the first-level variables, i.e., cognitive input, from teacher feedback, teaching attitude, learning resources, group cooperation, and teacher–student interaction. English knowledge generate level 1 variable, i.e., cognitive input.

**FIGURE 3:**
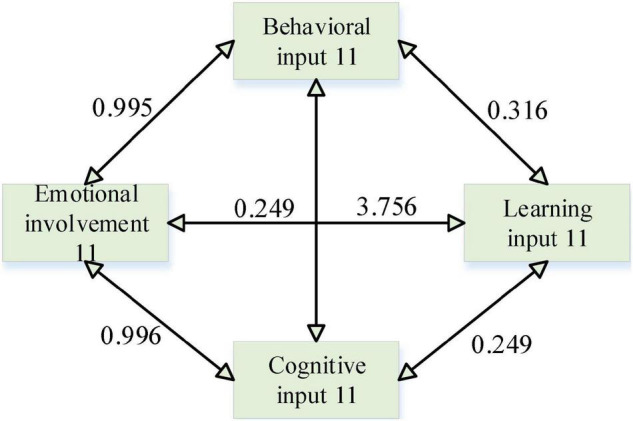
Path diagram of non-English majors.

In the path analysis of non-English majors’ English classroom learning engagement, the standardized path coefficient for behavioral engagement was 0.315 > 0, and the path showed a 0.01 level of significance (*z* = 3.472, *p* = 0.001 < 0.01), thus indicating that behavioral engagement has a significant positive effect on learning engagement. The standardized path coefficient of 0.249 > 0 for the effect of emotional engagement on learning engagement and this path showed a significance at the 0.05 level (*z* = 2.321, *p* = 0.020 < 0.05), thus indicating that emotional engagement has a significant positive effect on learning engagement. The standardized path coefficient was 0.436 > 0 for the effect of cognitive engagement on learning engagement, and this path was significant at the 0.01 level (*z* = 3.756, *p* = 0.000 < 0.01), thus indicating that cognitive engagement has a significant positive effect on learning engagement. Based on the above path analysis, a path relationship model of English classroom learning engagement of non-English majors was developed (Figure 3).

**FIGURE 4:**
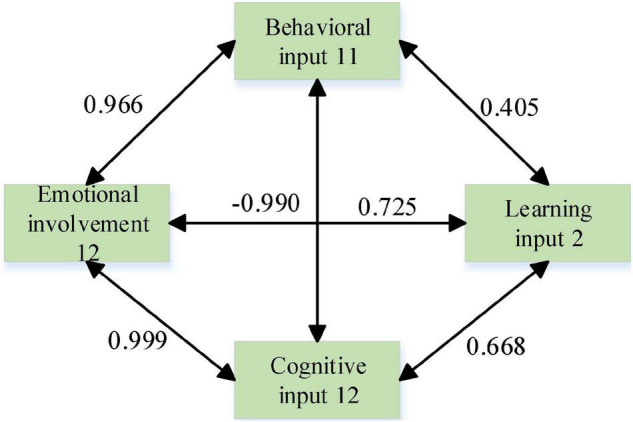
Path diagram of English majors.

In the path analysis of English majors’ English classroom learning engagement, the standardized path coefficient for behavioral engagement was 0.405 > 0 and the path showed a 0.01 level of significance (*z* = 16.533, *p* = 0.000 < 0.01), thus indicating that behavioral engagement has a significant positive effect on learning engagement 2. The standardized path coefficient of 0.668 > 0 for the effect of cognitive input on learning input was significant at the 0.01 level (*z* = 10.380, *p* = 0.000 < 0.01), thus indicating that cognitive inputs have a significant positive effect on learning input. This path did not show a significant effect of emotional engagement on learning engagement (*z* = −0.990, *p* = 0.322 > 0.05), thus indicating that emotional engagement does not have an impact on learning engagement. In addition, the author conducted a linear regression analysis with affective input, cognitive input, and behavioral input as independent variables and learning input as dependent variable, and the regression coefficient value of affective input was −0.036 (*t* = −0.589, *p* = 0.557 > 0.05), which also means that affective input does not have an effect on learning input. Based on the above path analysis, the path relationship model of English classroom learning engagement of English majors was established (Figure 4).

In summary, emotion, behavior, and cognition had significant path effects on learning engagement for non-English majors, but emotion did not have significant path effects on learning engagement for English majors.

## Application of Learner Emotion Recognition

The CNN structure and method proposed in this study can recognize learners’ emotions quickly and accurately, and support the direct input of camera-captured images as the original images, avoiding the feature extraction process in traditional recognition methods, and can recognize learners’ emotions in real time and quickly:

The learner model is an abstract representation of learner characteristics in the virtual learning environment, representing the learners that the learning environment can recognize and understand, and updating the learner characteristics information in real time, which is an important basis for implementing wisdom and personalization in the smart learning environment. Therefore, building a perfect learner model is the prerequisite and foundation for providing smart learning services to learners. At present, standards such as IEEE PAPI and IMSLIP have been established in the field of learner models, and researchers have also studied them from different perspectives. However, existing studies have mostly focused on basic information and cognitive-level attribute characteristics of learners, such as gender, age, knowledge level, cognitive ability, etc., and ignored or belittled factors such as learners’ context, preference, and emotion. The learner model should cover both basic information, academic information, relational information, and knowledge status of learners, as well as contextual characteristics and affective status. This study will contribute to the construction of a learner model for smart learning environments that incorporate affective features. The model mainly includes basic information, learner preferences, knowledge status, contextual characteristics, and affective states. Among them, basic information mainly includes name, gender, age, grade, contact information, etc.; learner preferences include learning style, content preference, interaction preference, media preference, etc.; knowledge state includes knowledge base, domain knowledge, mastery degree, etc.; contextual features include social context, cognitive context, technological context, etc. (Wang et al., 2018); affective state includes affective type and intensity, etc., and affective type and intensity will be determined by the learner emotion identification method proposed in this study will be obtained in real time after identification, so that the learner model can be updated and improved in time.

At present, research on smart learning environments mostly focuses on providing adaptive learning content according to learners’ knowledge levels, i.e., it emphasizes adaptive interaction at the cognitive level in smart learning environments, and less considers adaptive interaction of learners’ emotions. To provide more intelligent learning services, the adaptive interaction between the learning environment and learners is essential, and the fast and accurate real-time recognition of learners’ emotions is the basis for realizing the adaptive interaction of emotions in a smart learning environment. The emotion implied by the learning screen has a potential impact on learners’ learning interest, cognitive load and emotional state, and the emotion of the learning screen in a smart learning environment is an important expression of its emotional characteristics. This study will help realize harmonious emotional interaction in the smart learning environment by recognizing and judging learners’ emotional states based on their facial expressions when watching learning images, and automatically adjusting key visual emotional features of learning images by combining learners’ visual emotional preferences for learning images, including adjusting the background color and texture of the interface, enhancing the display of learning interest areas or key learning contents, adding hidden interesting Animation, etc., and fast, real-time, without affecting the normal online learning of learners, so that learners always maintain a positive, good emotional state.

## Conclusion

Accurate recognition of learner emotions is the key to achieving harmonious emotional interaction in a smart learning environment, and only by efficiently recognizing learner emotions can be the learning screen or intelligent teaching agent in a smart learning environment be adjusted accordingly to achieve self-adaptation and personalization. The traditional method of manual feature extraction followed by machine learning has been used for learner emotion recognition, which is complicated and less efficient. DL, as an important technology in artificial intelligence, takes the original image as input and learns autonomously by training the sample set, avoiding the explicit feature extraction process, with high performance and generalization ability. In addition, the down-sampling operation of the cooling layer enhances the robustness of the recognition algorithm.

This study proposes a DL-based learner emotion recognition method by classifying common learner emotions into normal, happy, angry, sad, frightened, focused, and board based on the existing research and building a large-scale learner emotion database. This study has encapsulated the learner emotion recognition program written by Matlab software into functions that can be called in C, C++, C#, Java, and other languages, in order to provide reference and help for theoretical researchers and platform builders of intelligent learning environments, and make contributions to the adaptive interaction of emotions in intelligent learning environments.

## Data Availability Statement

The original contributions presented in the study are included in the article/supplementary material, further inquiries can be directed to the corresponding author.

## Ethics Statement

The individual(s) provided their written informed consent for the publication of any identifiable images or data presented in this article.

## Author Contributions

DZ was responsible for designing the framework of the entire manuscript, from topic selection to solution to experimental verification.

## Conflict of Interest

The author declares that the research was conducted in the absence of any commercial or financial relationships that could be construed as a potential conflict of interest.

## Publisher’s Note

All claims expressed in this article are solely those of the authors and do not necessarily represent those of their affiliated organizations, or those of the publisher, the editors and the reviewers. Any product that may be evaluated in this article, or claim that may be made by its manufacturer, is not guaranteed or endorsed by the publisher.
